# Abstract of the Proceedings of the Buffalo Medical Association

**Published:** 1870-08

**Authors:** Wm. C. Phelps

**Affiliations:** Secretary


ART. III.—*Abstract of the Proceedings of the Buffalo Medical Association.*

BUFFALO, AUGUST 2, 1870.

The Vice-President, Dr. THOMAS M. JOHNSON, in the chair.

Members present—Drs. WHITE, MINER, JOHNSON, ROCHESTER, GAY, BARNES, SAMO, POTTER, GOULD, PHELPS.

The minutes of the last meeting of the Association were read and approved.

The application of Dr. GEORGE D. SLOCUM for membership was received, and under the rule, laid over till the next meeting.

Dr. BARNES read the following report of a case of retro uterine hæmatocle, which was operated on by Prof. WHITE, on the 28th of May last.

The subject of the operation was a Mrs. FRY, aged 34, a resident of this city.

The previous history of the patient, so far as it relates to the disease in question, is as follows:—She had been accustomed to hard work, and much exposure to vicissitudes of temperature. She had been married nearly twenty years, and had given birth to six children. Her last labor, two years ago, had been an unusually protracted one, but finally terminated, through the efforts of nature, in the birth of a dead child. Previous to this occurrence her health had been remarkably robust, but was less so afterwards.

One year ago, at a menstrual period, after exposure to cold, she was seized with very severe pains, of an expulsive nature, so characteristic that both she and her medical attendant thought at first that she was about to abort; but no clots were passed, nor was there other evidence of the expulsion of a product of conception.

From this time forward her health rapidly and progressively failed. The menses recurred at uncertain intervals, and the flow was diminished in quantity and duration; a sense of fullness and weight, which finally became oppressive, was experienced in the pelvic region, together with tenderness and more or less constant pain, which was increased in walking. The abdomen became notably enlarged; sympathetic derangements of the digestive system ensued, frequent nausea and vomiting, defective appetite and assimilation of food, with rapid emaciation and loss of strength. There was also mechanical interference with the performance of the functions of the rectum and bladder. From these causes the patient finally became so reduced as to be unable to leave her bed. It was in this condition—when she had just passed a period of flowing lasting seven weeks—that she was first seen by Dr. White.

An external examination showed the abdomen to be enlarged, the upper portion somewhat tympanitic, while the hypogastric and right iliac regions were occupied by a tumor, which could be easily felt by the hand. A vaginal examination disclosed a large, smooth rounded tumor, moderately firm in consistence, descending to and occupying the pelvic brim. The finger could be readily passed around this tumor posteriorly, but not around it on its right side, where it seemed to extend over into the iliac region. The uterus was displaced forward and upwards, so that no trace of it could be discovered, except by passing the finger up closely under the symphysis pubis, when the os uteri could with some difficulty be recognized. The fundus of the uterus could be felt above the pubic bone. A rectal examination still further disclosed the shape and consistence of the tumor.

The diagnosis was retro uterine hæmatocle, and it was determined to evacuate the tumor by puncture through the vagina. The wall of the sack was first pierced by an exploring trochar, from which there issued drops of thick, dark blood, at once confirming the diagnosis. A full sized trochar and canula was next introduced, and the trochar being drawn, a large quantity of the same dark blood passed by the channel thus furnished. A small flexible bougie was then passed through the canula into the cavity of the tumor, and over this the canula was withdrawn, leaving the bougie in position. With this as a guide, a pair of long bladed forceps was next inserted, closed, as deeply as possible, and the blades being then separated, and firmly held, the instrument was drawn quickly out, enlarging the opening in its exit by laceration, so that a finger could be introduced, and a large quantity of clotted blood removed. A still further enlargement of the opening by the knife was deemed necessary, after which the operation was concluded by breaking down and removing the remaining clots. More than twenty ounces in all were taken from the sac.

A flexible catheter was inserted into the wound, and fastened to the thigh to facilitate drainage, with instructions to remove in case it produced irritation. A compress was laid upon the abdomen, and a bandage applied as after parturition. Half a grain of morphine was given, to be followed by the same quantity in six hours.

The subsequent history of the case is briefly as follows: On the day following the operation the patient presented a favorable appearance. There had been a liberal drainage of dark colored blood during the night. The cavity was gently but freely washed out with tepid water, by means of a Davidson's syringe, with long tube, which brought away considerable clotted blood. Pulse 80. The diet was not limited by instructions, but she took beef tea, rice and milk. On the third day, the discharge having become slightly fetid, the Liq. Sodæ Chlorinat was added to the water used to clean the cavity, and the same was continued until the discharge ceased. As the patient complained of the canula, it was removed. On the fifth day the bowels were moved by an enema. On the ninth day, a vaginal examination showed that the large tumor had disappeared, its anterior wall having so far receded that the extremity of the finger, with difficulty, reached the now greatly contracted orifice, through which the contents of the tumor had escaped. The discharge had become small in quantity, thin, and nearly colorless. The uterus had resumed its normal position, and the rectum and bladder performed their functions spontaneously, and without pain. The speculum, which could now bring the cervix plainly into view, disclosed the existence of a chronic inflammation of the membrane lining the neck and body of the womb. The patient could walk a little about the room, and on the thirteenth of June, the sixteenth day after the operation, she walked to my office, a distance of more than half a mile.

The above case is a good example of a form of disease which, although no longer new to the profession, is not yet so generally familiar as to be without interest.

The tumor is caused by an effusion of blood, either into the peritoneal cavity, or externally to the peritoneum, into the loose tissue connecting the pelvic organs. A ruptured ovary is most frequently the source of the hemorrhage, though it may come from any of the pelvic vessels, or by flowing backwards from the womb, may escape from the fimbriated extremity of the fallopian tube. Anything causing extreme pelvic congestion or mechanical obstruction to the menstrual function may determine the effusion.

Dr. White would only add to the above narration that the tumor was mostly confined to the upper part of the pelvis. This was also the fact in one of his earlier cases, that of a woman from St. Louis, upon whom he performed a similar operation. He much prefers the vagina to the rectum, as the channel through which to reach the tumor. An experience of twenty-four cases has convinced him that when the operation is made through the rectum it is very likely to be followed by a fistula, and the sack also may become filled with fecal matter. The operation is somewhat more difficult to perform, and presents no advantages over that through the vagina.

The diagnosis is attended with no difficulty when the trocar is used, as it always was by him. Without it much difficulty would be experienced, and a pôsitive knowledge in some cases met with, would be impossible.

The sack is filled with blood, more or less mixed with other fluids, and sometimes with serum alone, as occurred in the case of a patient from Pennsylvania, which proved to be an ovarian cyst which had fallen down behind the womb, and had not been attended with inflammation. This case was treated by evacuating the serum, and by injecting tr. iodine into the sack with a good result.

In some patients, as in the case which Dr. BARNES has related, the os-uteri does not present itself to the speculum, and cannot be seen.

The tumor should be opened as soon as discovered. Having with Simpson's large trocar evacuated all that can be passed through, it should be withdrawn, and some instrument, as the forceps, passed into the sack, and, the blades being open, drawn out so that the opening may be enlarged by being torn. The sack can then be entirely emptied, and its ragged edges will prevent the opening from closing as soon as it otherwise would. Should it close, the operation must be repeated, as occurred in a patient on whom he operated for Dr. Fuller, of Le Roy, the wound having closed from the after directions not being perfectly understood.

Dr. ROCHESTER related a case of retro uterine tumor which came to him, in which the exploring needle was used, which led him to suppose the tumor to be fibrous, being as hard as scirrhus growth; it interfered greatly with defecation on account of its size and hardness. She was taken to the hospital, and suppuration took place, with great relief. He again saw her with Dr. Brown, and could still detect a hard, large tumor. The needle was introduced, and a pint and a half of glairy fluid drawn out; and the cavity was injected with carbolic acid in the proportion of two grains to the ounce. Dr. R's experience of twelve cases led him substantially to agree with the treatment narrated this evening, and would lay great stress on the large opening, and would use no tube, as it is often followed by great irritation.

A fistula resulted in a case under his care, which remained undisturbed, as the patient would not submit to any treatment. Eight months of good health, however, cured the fistula.

Dr. MINER said this subject had before occupied the attention of the association, and been pretty thoroughly discussed. It was a well-established fact that collections of pus in the pelvic cellular tissue generally if they burst spontaneously, burst into the bowel. This had happened in three cases that had come within his knowledge: it was the exception to the rule when they opened spontaneously into the vagina. Following the indications of nature, thus plainly manifest, he had opened *such* collection by way of the rectum, and with good result; no fistula followed, and no accumulation of fecal matter in the emptied sack was noticed. It had some advantages, the most notable being the retention by the splincter of the pus in the bowel after it had left the sac, where it can remain until emptied from time to time, as in defecation, thus being much more cleanly and comfortable to the patient than is the case when discharging through the vagina. He did not mean to say that he would always operate through the rectum, but if the fluid collected be pus, and could as well be evacuated that way as any other, he would prefer it. In haematocle, he would prefer the vagina to the rectum, as he might not feel warranted in making as large an opening in the bowel, as would sometimes be necessary in such cases.

Dr. ROCHESTER said that even collections of pus do not always open into the bowel, as he saw in consultation with Dr. MACK, of St. Catharines, in which it found exit through the walls of the abdomen.

Dr. MINER said that he would not wait for spontaneous evacuation of pus, but only wished to express his opinion as to the best way of evacuating it.

Dr. ROCHESTER, in presenting two gall stones to the examination of members present, said that they were taken from the body of Mr. Thomas E. Young, at a *post mortem* examination. The patient died after an illness of four days of localized peritonitis, resulting from inflammation of the *appendix vermiformis*, caused by the gall stones which I have exhibited. Perforation occurred twenty-four hours before death, and one of these stones was found at the opening in the appendix. I look on this case with interest, as it confirms an opinion which I have for years held, that the foreign bodies which are so frequently said to have caused death by slipping into the appendix are not seeds, pits, &c., &c., but are gall stones, and consist as does this one of cholestein. Rokitansky says that the disease in the outset is a catarrhal affection, which dilates the orifice, and allows the gall stones to fall into the appendix.

Dr. MINER said he was deeply interested in the *post mortem* examination of the case related, which he was favored with the opportunity of witnessing. He had admired the sagacity which Professor Rochester had shown in being able to give just as full and perfect an account of the causes and conditions of Mr. Young's death before, as after, the examination. He asked if it was not common to find foreign substances in the appendix, which were not the cause of inflammation,—if they did not, sometimes, at least, remain harmless? He noticed the opening into the apendix in this case was very small, and did not appear likely to admit any foreign substance, unless it was forced into it; was surprised at the minuteness of the orifice through which such substances had passed.

Dr. ROCHESTER explained that Rokitansky says that as a result of the catarrhal affection, the opening from the colon to the appendix becomes unnaturally large, and at such times the stones gain access to the appendix. Inflammation is then set up, which closes the opening by swelling, and this is its condition at death, which explains the smallness observed at *post mortem* examinations. This opinion is supported by occurrence of the diarrhœa which such cases, as a rule have, for some time previous, been liable to. In the case of Mr. Young this was a well marked symptom, as he had a diarrhœa for ten days previous to the peritonitis. Rokitansky had, he believed described cases where similar substances had apparently remained harmless.

Dr. WHITE wished to bear testimony to the accuracy and acuteness of Dr. Rochester in the diagnosis of disease of the appendix, of which he had seen many proofs before the present case. He had seen him express a positive opinion in obscure cases, as though the walls of the abdomen were transparent, and these opinions had always been confirmed by *post mortem* examinations.

Diarrhœa, infantile diseases and disorders of the bowels were reported as prevailing.

Dr. MINER wished to call attention to the proper food for infants. He had lately observed a great deal of suffering in infants, from all sorts of food, which, in his opinion, added largely to the mortality of that class of patients, such as sago, tapioca, rice, crackers, corn starch, oatmeal, &c., and was of opinion that in almost all cases where the mother's milk was not sufficient, cow's milk and water should alone be substituted.

Dr. White said that Dr. Miner had opened an important subject, and hoped that some evening would he devoted to the consideration of it. He would say that mother's milk, when present, should be the great bulk of the child's nourishment. When artificial food must be relied on, cow's milk, diluted about one half with water, and sweetened with sugar, is the proper food. No farinaceous article of diet should be given.

Adjourned.

WM. C. PHELPS, Secretary.

